# A Patient-Matched Entire First Metacarpal Prosthesis in Treatment of Giant Cell Tumor of Bone

**DOI:** 10.1155/2017/4101346

**Published:** 2017-06-15

**Authors:** Thipachart Punyaratabandhu, Boonrat Lohwongwatana, Chedtha Puncreobutr, Arkaphat Kosiyatrakul, Puwadon Veerapan, Suriya Luenam

**Affiliations:** ^1^Department of Orthopaedics, Phramongkutklao Hospital and College of Medicine, Bangkok, Thailand; ^2^Department of Metallurgical Engineering, Faculty of Engineering, Chulalongkorn University, Bangkok, Thailand

## Abstract

Giant cell tumor of the bones occurring in the first metacarpals frequently requires entire metacarpal resection due to the aggressive nature and high rate of recurrence. Bone reconstruction can be performed with autogenous bone grafts. Here we describe a new technique of reconstruction using a patient-matched three-dimensional printed titanium first metacarpal prosthesis. This prosthesis has a special design for ligament reconstruction in the proximal and distal portions. Good hand function and aesthetic appearance were maintained at a 24-month follow-up visit. This reconstructive technique can avoid donor-site complications and spare the autogenous bone grafts for revision options.

## 1. Introduction

Giant cell tumor (GCT) of bones or osteoclastoma is a benign locally aggressive tumor. Metacarpals are uncommon sites of occurrence, accounting for 1% to 5.5% of cases [1–6]. Metacarpal GCT always displays more aggressive behavior and affects younger patients than GCT in other sites [3, 4]. Based on our literature review, the first metacarpal is most frequently affected [4–6]. At the time of presentation, the tumor usually exceeds 3–6 cm in length and bulges beyond the confines of the cortex, giving an expanded contour without periosteal reaction. GCT of the first metacarpal also has the high possibility of recurrence [2, 7, 8]. The local recurrence following curettage, with or without bone grafting, has been reported to be as high as 90% [2, 3]. For this reason, many authors had recommended the extensive en bloc resection as the standard treatment [2, 9, 10]. Following the resection, replacement of the bone defect with autogenous bone graft or allograft is essential in order to maintain thumb function. In the present report, we describe a new reconstructive technique using a patient-matched entire first metacarpal titanium prosthesis which was created with 3D printing technology.

## 2. Case Report

**Figure 1:**
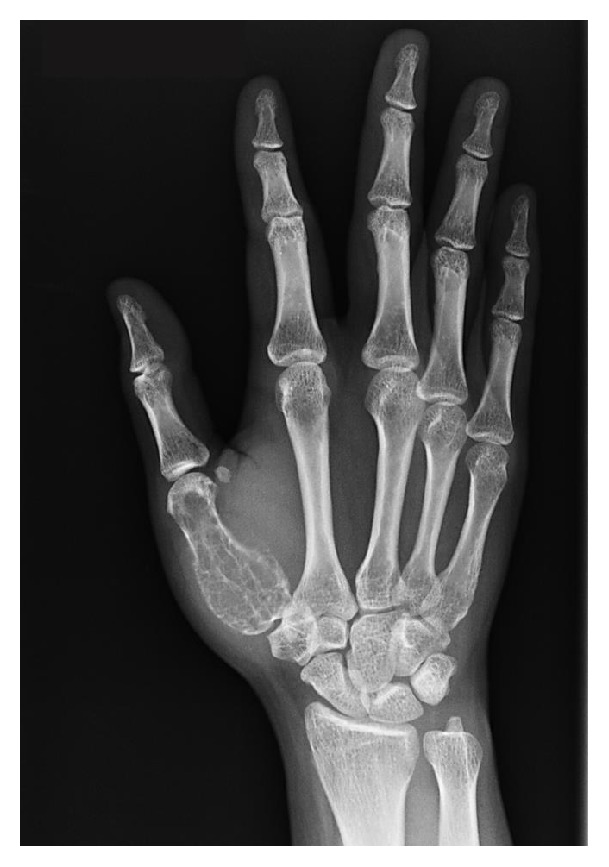
Plain radiograph showing expansile osteolytic lesion involving the entire length of the first metacarpal.

**Figure 2:**
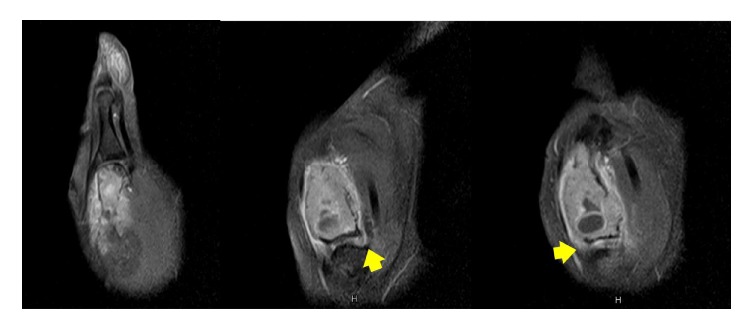
MRI showing the extension of the tumor into the surrounding soft tissue and around the first carpometacarpal joint (yellow arrow).

**Figure 3:**
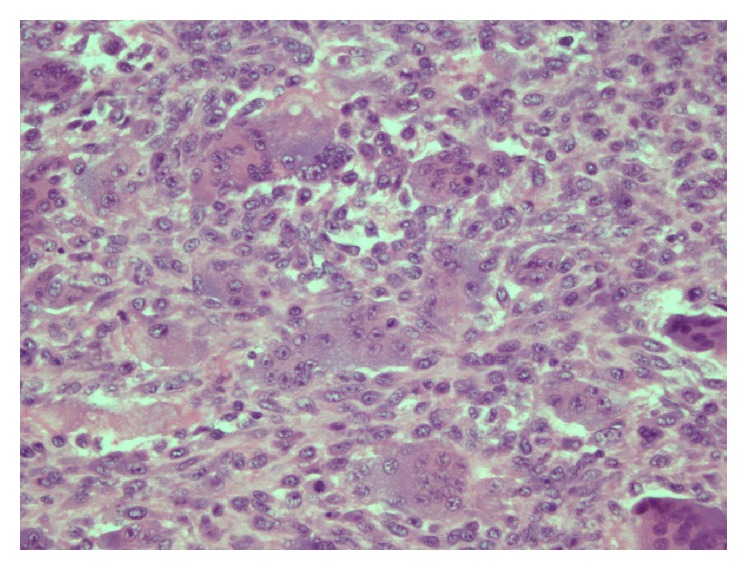
Photomicrograph of tumor histology demonstrating many osteoclast-like giant cells in a background of numerous round-to-spindle shaped mononuclear cells.

A 37-year-old female presented to our clinic with progressive painful swelling and restricted movement of the right thumb for a duration of 4 months. Radiographs revealed an expansile osteolytic lesion involving the entire length of the first metacarpal bone (Figure 1). The MRI demonstrated irregular expansion of the tumor breaking through the cortex. Extension of the tumor into the surrounding soft tissue and around first carpometacarpal joint was observed (Figure 2). The pathological findings from the core needle biopsy were consistent with the GCT of bone (Figure 3).

**Figure 4:**
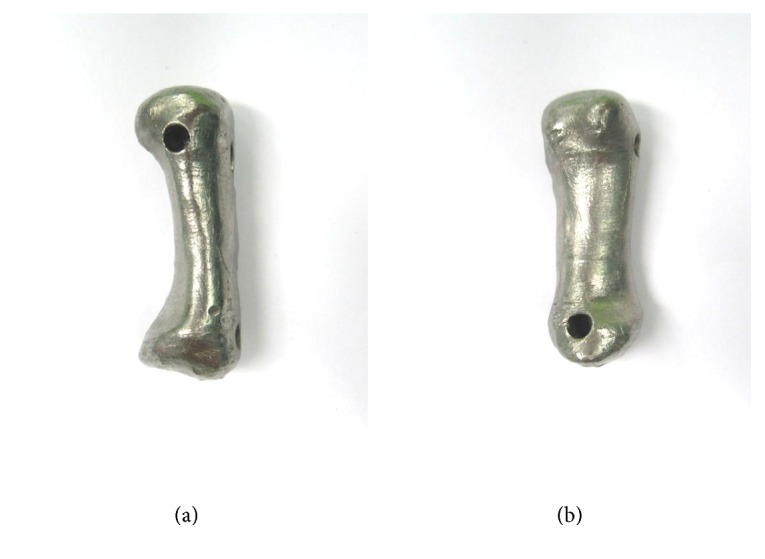
Photographs of the prosthesis before implantation: (a) anterior aspect and (b) volar aspect.

The en bloc resection was performed. The first metacarpal and the trapezium were excised, and the defect was temporarily bridged with bone cement. Six months later, a repeat MRI revealed no evidence of tumor recurrence. After a discussion with the patient regarding the reconstructive planning, she disallowed any option of using her autogenous bone grafts. A surgical treatment with patient-specific prosthesis was subsequently offered. A computed tomography scan of the patient's left metacarpal was done and used as a mirror image to create the custom mold by an Electron Beam Melting 3D printing technique. This mold was subsequently used to cast the entire titanium prosthesis. Multiple holes were designed in the proximal and distal portions of the prosthesis for the ligament reconstruction and temporary fixation (Figure 4).

**Figure 5:**
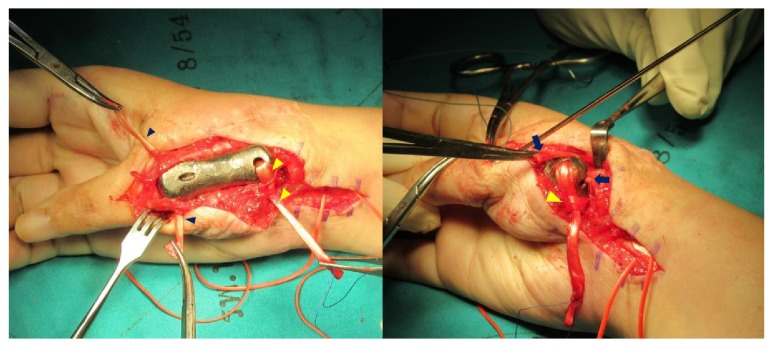
Intraoperative photographs showing 3D printed titanium first metacarpal prosthesis with the ligament reconstruction in the proximal and distal portions: free palmaris longus tendon graft (blue arrow head), flexor carpi radialis tendon (yellow arrow head), and extensor pollicis brevis tendon (blue arrow).

Intraoperatively, an incision was made over the dorsal aspect of the thumb metacarpophalangeal (MCP) joint, coursed along the radial insertion of the thenar muscles, curving ulnarly along the distal wrist crease, and extending longitudinally over the flexor carpi radialis (FCR) and palmaris longus (PL) tendon. The superficial branches of the radial nerve and artery were identified and protected. The biomembrane encapsulating the cement spacer was incised longitudinally. Once exposed, the cement spacer was removed and replaced by the titanium prosthesis. The ligament reconstruction was performed (Figure 5). The collateral ligaments and dorsal capsule of the MCP joint were reconstructed with a free PL tendon graft. The PL graft was harvested using a tendon retriever. The graft was passed through a premade distal side hole of the prosthesis centered over the origin of radial and ulnar collateral ligaments. Each end of the graft was woven through and sutured with the remaining of the collateral ligament which was still attached to the insertion on proximal phalanx. The residual graft was folded over the dorsal aspect of the MCP joint and sutured to itself at the exiting point on the contralateral side in order to reform the dorsal capsule. A 1.6 mm K-wire was inserted from the distal dorsal premade hole of the prosthesis to the proximal phalanx to maintain the MCP joint in 20 degrees of flexion. The proximal portion of the prosthesis was also stabilized by the ligament reconstruction. The remnant of extensor pollicis brevis (EPB) tendon from the previous operation was inserted through the proximal oblique hole entering from the articular surface and exiting on the posterior aspect of metaphysis. A small transverse incision was made over the FCR tendon 8 cm proximal to the distal wrist crease. The radial half of the FCR tendon was harvested as a distally based graft. The free tendon end was delivered to the distal wound and passed through a premade proximal sagittal hole of the prosthesis from the volar edge to the dorsal aspect. The exiting graft was brought anteriorly to loop around the ulnar half of FCR tendon and folded backward to wrap around EPB tendon. The metacarpal was held in 40 degrees of palmar abduction and 20 degrees of radial abduction. Both FCR and EPB grafts were pulled snuggly to stabilize the prosthesis and sutured to themselves. The remainder of grafts were folded and filled in the trapezial space to act as a soft tissue interposition. The biomembrane was closed with absorbable suture.

**Figure 6:**
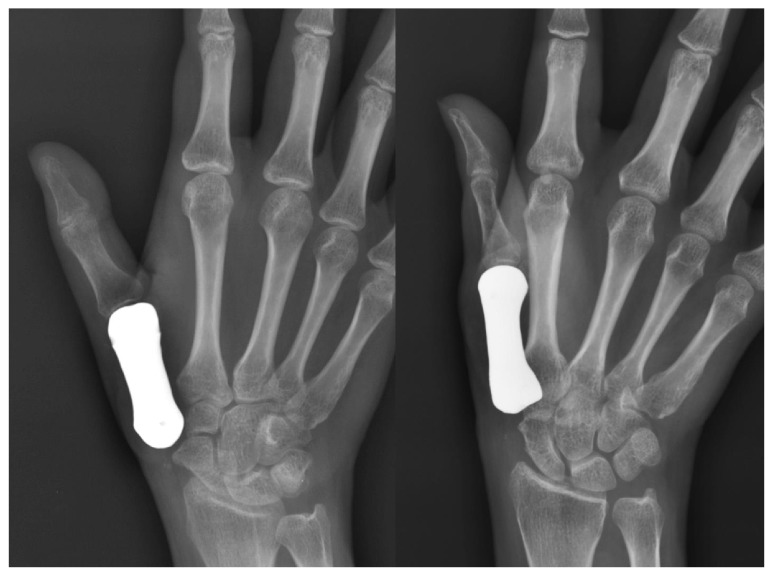
Plain radiographs of a patient-matched total first metacarpal prosthesis.

**Figure 7:**
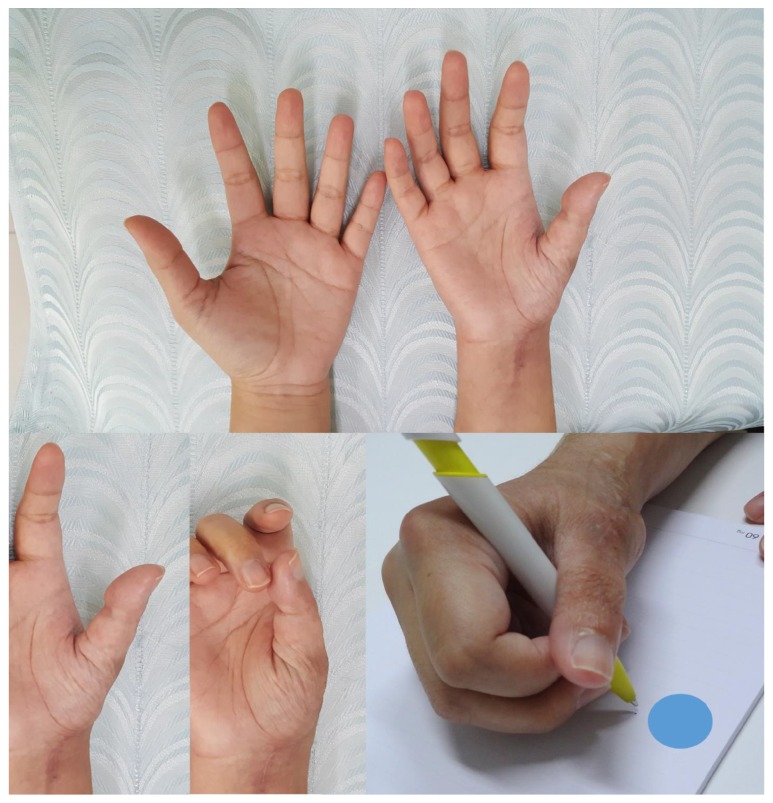
Postoperative appearance and range of motion at 24 months postoperatively.

Postoperatively, the hand was placed in the thumb spica cast for 4 weeks after which the cast was switched to a thumb spica removable splint for an additional 2 weeks. The K-wire was removed at 12 weeks under local anesthesia. At the latest follow-up, 2 years after surgery, the patient has no pain and there are no signs of tumor recurrence. Five mm shortening of the thumb was observed. The MCP and CMC joints were stable both clinically and radiologically (Figure 6). The patient was able to touch the tip of the middle finger with the tip of her thumb (4 points on the Kapandji thumb opposition scores). The range of motion of MCP joint was 30 degrees of flexion and 0 degrees of extension. The range of motion of CMC joint was 5 degrees of flexion, 25 degrees of extension, and 45 degrees of abduction (Figure 7). The grip strength was 9 kg (27 kg on the contralateral side) and the key-pinch strength was 2 kg (7 kg on the contralateral side) measured using the Jamar dynamometer. Although the range of motion and strength was diminished compared with the normal side, the patient was still satisfied with the cosmetic and functional outcomes.

## 3. Discussion

GCT of the first metacarpal bone always presents in the advanced stage, and surgical treatment with an extensive en bloc resection of the entire metacarpal is often required [2, 9, 10]. The reconstructive operation in this area is very challenging as the thumb has a fundamental role in nearly all grasping and handling maneuvers. Loss of thumb function imparts a 40% to 50% rate of impairment to the upper extremity [11]. In the present study, we describe the novel technique of reconstruction using a patient-matched 3D printed titanium metacarpal prosthesis.

The prosthesis in the present study had been manufactured to mimic the original first metacarpal bone using 3D printing technology. The prosthesis can be designed to have the same length as the original, and the articular section was anatomically contoured for the line to line contact with the corresponding articular surface. However, a proximal articulating surface was not used due to the loss of the trapezium. The soft tissue reconstruction and tendon interposition were performed to stabilize the CMC joint and maintain the proper length of thumb. The FCR tendon was passed through the proximal sagittal premade tunnel for reconstruction of the deep anterior oblique ligament and dorsal radial capsular ligament which are the key stabilizers of the CMC joint. The EPB tendon was inserted through the proximal posterior oblique tunnel for the posterior ligament reconstruction to create the force couple and maintain the prosthesis in an appropriate alignment. After the surgery, the prosthesis remained stable clinically and radiographically at the final follow-up. Preserving the mobility at the MCP and CMC joints may provide benefit for increasing the thumb range of motion and the ability of grasping and pinching.

First metacarpal reconstruction with autogenous bone grafts from the iliac crest, fibula, and radius has been described previously [3, 5, 6, 12]. The additional procedures involved with harvesting the bone graft increases the risk of donor-site morbidities. Fusion of both MCP and CMC joints is always necessary to maintain the thumb in a position of function [6]. For this reason, the range of the thumb motion is inevitably significantly restricted. Autogenous metatarsal transfer as an osteochondral graft has been proposed in order to increase the thumb motion by preserving the movement at MCP and CMC joints [13]. However, mismatch of the joint surfaces and bone length has been reported [5], and another reconstructive procedure for the metatarsal defect was needed. Alternatives other than autogenous grafts include allograft [14] and contoured bone cement [15]. However, the information regarding the outcomes of these options is limited and only published in single-case reports.

The advent of 3D printing technology is revolutionizing orthopedic reconstructive surgery [16, 17]. Based on the symmetry of the human skeleton, we can reverse the 3D images of the normal bone to the contralateral side in order to manufacture the anatomic prosthesis for the missing part [17]. Although this technology is now widely accessible and affordable, only few articles regarding the patient-specific 3D printing-based prosthesis have been published in medical journals to date [16]. The commonly used metal materials in the patient-specific prostheses include titanium, cobalt-chromium alloy, and stainless steel [18]. Titanium had been chosen in the present study because of the high biocompatibility, good mechanochemical properties, and light weight nature [19].

In conclusion, a patient-matched metacarpal titanium prosthesis appears to provide satisfactory functional outcome and aesthetic appearance. This could be a valid alternative for the treatment of the entire first metacarpal bone loss following the en bloc resection. Although the follow-up period is not sufficiently long enough to commit to longevity and retention of function, this reconstructive technique has the benefit of avoiding the donor-site complications and sparing the autogenous bone graft for the revision options.
